# Supplementary material for: Effect of phyB and phyC loss-of-function mutations on the wheat transcriptome under short and long day photoperiods
Source: BMC Plant Biol. 2020 Jun 29;20:297. doi: 10.1186/s12870-020-02506-0 (PMC7325275; doi:10.1186/s12870-020-02506-0)
Supplement: Supplementary file 1 — Additional file 1. Figs. S1-S5, Tables S1-S8. [file 12870_2020_2506_MOESM1_ESM.pdf]

Effect of *phyB* and *phyC* loss-of-function mutations on the wheat transcriptome under short and long day photoperiods

Additional file 1. Tables and figures in this additional file:

Figure S1: Heading date of *phyB* and *phyC* mutants in SD photoperiods. For each phytochrome gene, data is presented for plants carrying non-functional copies of only the A homeolog (-A), only the B homoeologue (-B) or both A and B alleles combined (null). Experiments were performed in the tetraploid variety 'Kronos' (Genomes AABB).

Figure S2: Representative plants of WT, *phyB*-null and *phyC*-null genotypes at eight-weeks of age. SD RNA-seq data was generated from leaf tissue harvested at this stage.

Figure S3: Transcript levels (TPM) from RNA-seq data of selected flowering time genes in wild-type, *phyB*-null and *phyC*-null plants grown in SD conditions. *FT-A3* transcripts were not detected in the RNA-seq experiment and are not displayed here. *VRN1* genes are displayed in a separate panel. Error bars represent standard error.

Figure S4: MDS plot for all SD and LD RNA-seq samples. LD data consists of two experimental replicates each with four biological replicates of each genotype. SD data consists of one experimental replicate.

Figure S5: Number of DE genes between SD and LD samples of each genotype. SD samples were harvested at 8 weeks of age and consisted of 4 biological replicates. LD samples were harvested at 4 weeks of age and consisted of 8 biological replicates.

Table S1: Summary of RNA-seq reads and mapping rates from SD samples.

Table S2 – Top 10 significant enriched GO terms among genes differentially regulated by *PHYB,* by *PHYC,* and by both *PHYB* and *PHYC* in SD conditions.

Table S3: Summary of RNA-seq reads and mapping rates from LD samples.

Table S4 – Top 10 significant enriched GO terms among genes differentially regulated by both *PHYB* and *PHYC* under different photoperiod conditions.

Table S5: Selected genes regulated by both *PHYB* and *PHYC* in both SD and LD photoperiods. Fold change between WT and respective *phy*-null mutants are presented. The values for LD are the mean fold-change in expression between both experimental replicates. a - *FT-A1* and *FT-B1* expression was zero in the *phy*-null mutants in these comparisons.

Table S6: Selected genes regulated by both *PHYB* and *PHYC* in SD photoperiods but not LD photoperiods. Fold change between WT and respective *phy*-null mutants are presented as the mean fold-change between both experimental replicates. a - *FT-A4* expression was zero in the *phy*-null mutants in these comparisons.

Table S7: Selected genes regulated by both *PHYB* and *PHYC* in LD photoperiods but not SD photoperiods. Fold change between WT and respective *phy*-null mutants are presented as the mean fold-change between both experimental replicates.

Table S8: *PHYB*- and *PHYC*-regulated splicing-related genes. Mean TPM and fold-change for each genotype was calculated from four biological replicates in SD samples, and eight biological replicates for LD samples.

Figure S1: Heading date of *phyB* and *phyC* mutants in SD photoperiods. For each phytochrome gene, data is presented for plants carrying non-functional copies of only the A homeolog (-A), only the B homoeologue (-B) or both A and B alleles combined (null). Experiments were performed in the tetraploid variety 'Kronos' (Genomes AABB).

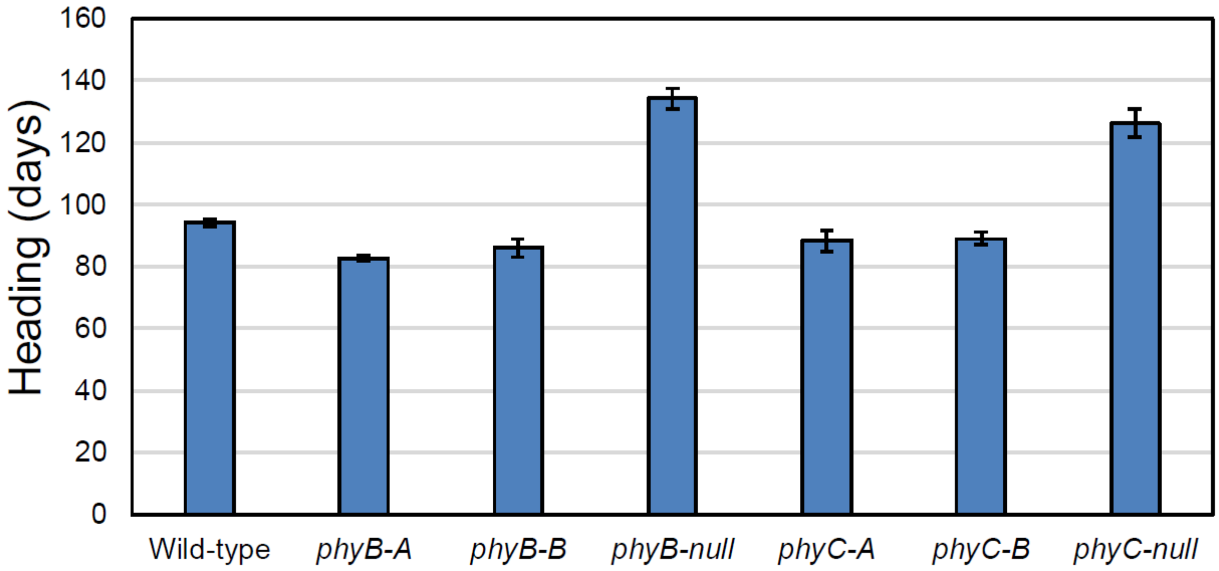

Figure S2: Representative plants of WT, *phyB*-null and *phyC*-null genotypes at eight-weeks of age. SD RNA-seq data was generated from leaf tissue harvested at this stage.

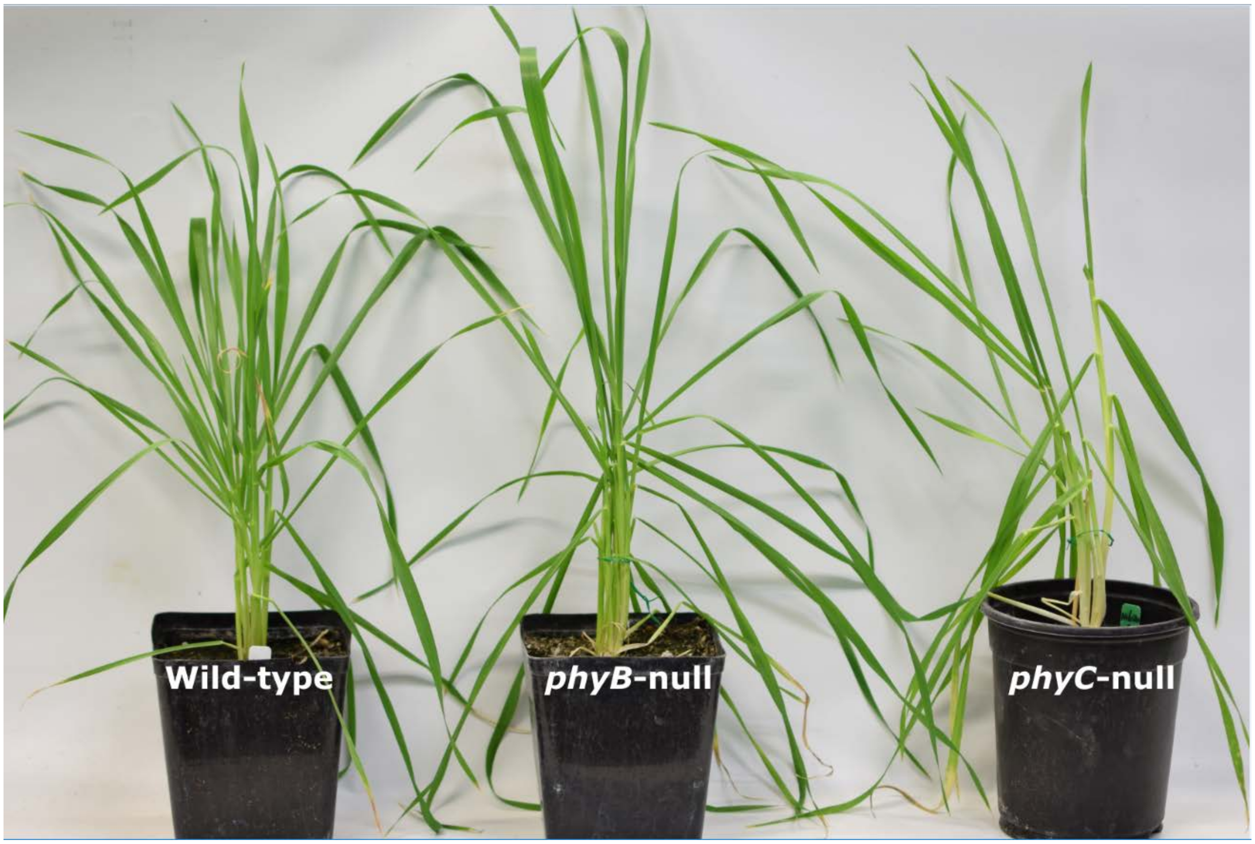

Figure S3: Transcript levels (TPM) from RNA-seq data of selected flowering time genes in wild-type, *phyB*-null and *phyC*-null plants grown in SD conditions. *FT-A3* transcripts were not detected in the RNA-seq experiment and are not displayed here. *VRN1* genes are displayed in a separate panel. Error bars represent standard error.

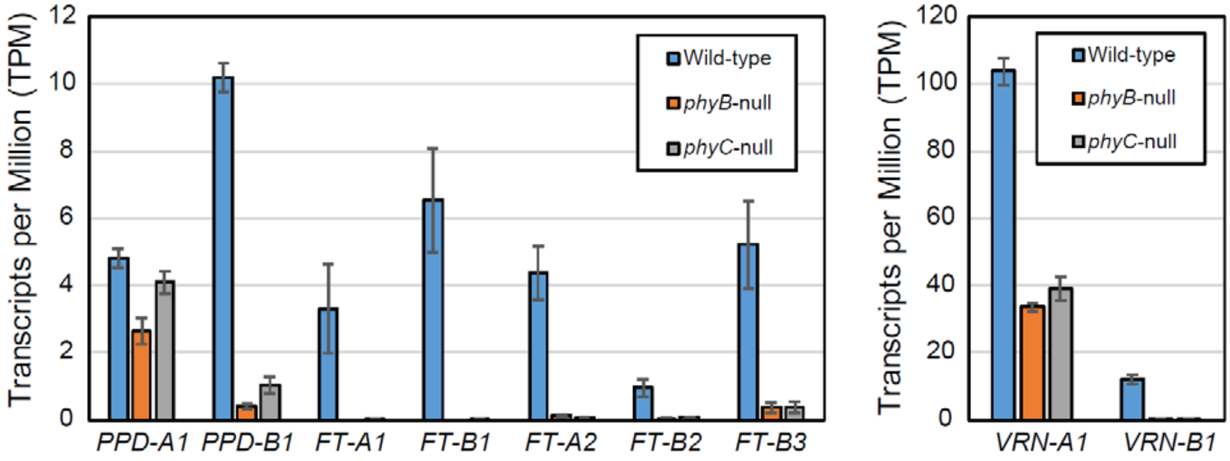

Figure S4: MDS plot for all SD and LD RNA-seq samples. SD data consists of four biological replicates of each genotype and one wild-type sample was used as a control for both mutants. LD data consists of two experimental replicates each including four biological replicates of *phyB*-null, *phyC*-null and wild-type sister lines for each respective mutant.

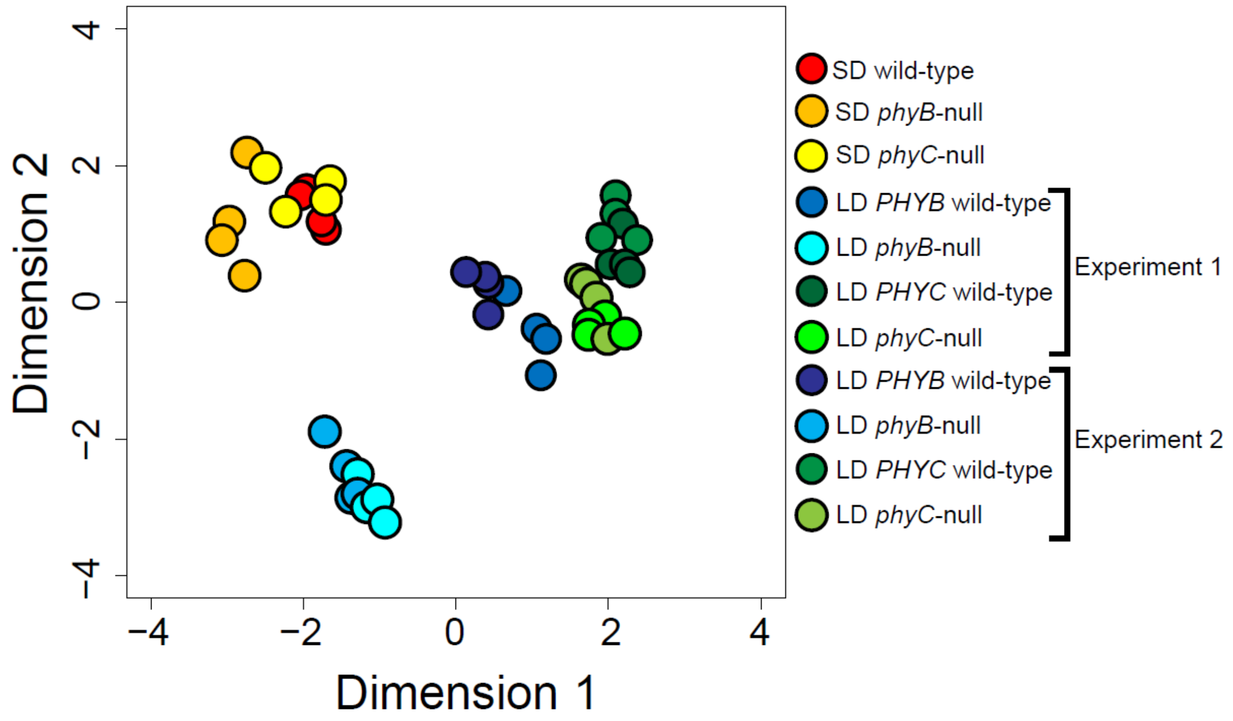

Figure S5: Number of DE genes between SD and LD samples of each genotype. SD samples were harvested at 8 weeks of age and consisted of 4 biological replicates. LD samples were harvested at 4 weeks of age and consisted of 8 biological replicates.

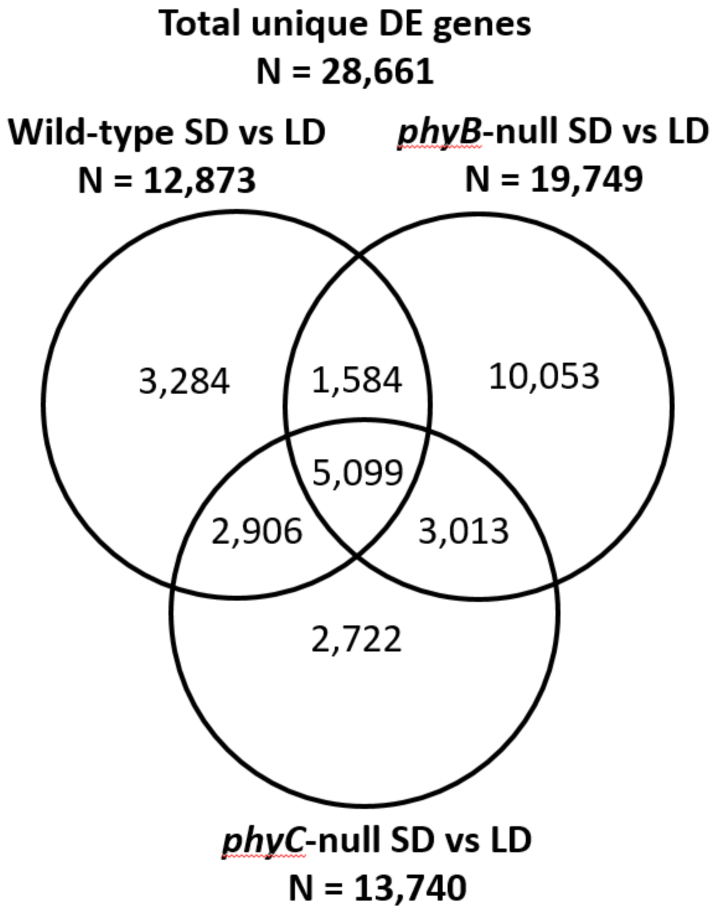

Table S1: Summary of RNA-seq reads and mapping rates from SD samples.

Genotype	Biological replicate	Total raw reads	Reads discarded		Total trimmed reads	Uniquely mapped reads	
			N	%		N	%
**Wild type**	1	58,502,128	1,013,691	1.7	57,488,437	39,607,623	68.9
	2	58,830,113	1,108,319	1.9	57,721,794	40,219,742	69.7
	3	67,577,803	1,139,114	1.7	66,438,689	45,702,198	68.8
	4	65,430,265	1,191,428	1.8	64,238,837	44,160,635	68.7
***phyB*-null**	1	54,007,718	837,760	1.6	53,169,958	37,368,473	70.3
	2	63,069,027	1,079,413	1.7	61,989,614	43,188,697	69.7
	3	64,820,390	1,047,399	1.6	63,772,991	44,409,244	69.6
	4	66,273,908	1,174,901	1.8	65,099,007	45,971,041	70.6
***phyC*-null**	1	66,985,324	1,317,576	2.0	65,667,748	45,956,376	70.0
	2	66,376,725	1,289,943	1.9	65,086,782	45,148,294	69.4
	3	86,487,404	1,503,903	1.7	84,983,501	58,948,262	69.4
	4	72,794,612	1,384,225	1.9	71,410,387	49,720,482	69.6
**Mean**		65,929,618.08	1,173,972.67	1.8	64,755,645	45,033,422	69.7

Table S2 – Top 10 significant enriched GO terms among genes differentially regulated by *PHYB*, by *PHYC*, and by both *PHYB* and *PHYC* in SD conditions.

***PHYB*-regulated**					
**GO ID**	**Term**	**Annotated**	**Significant**	**Expected**	***P***
GO:0055114	Oxidation-reduction process	4,000	540	396.1	5.30E-15
GO:0006468	Protein phosphorylation	3,908	529	387.0	6.60E-15
GO:0044710	Single-organism metabolic process	9,737	1,161	964.1	8.50E-15
GO:1903825	Organic acid transmembrane trans.	244	64	24.2	2.30E-13
GO:1905039	Carboxylic acid transmembrane trans.	237	61	23.5	2.00E-12
GO:0015849	Organic acid transport	324	75	32.1	2.10E-12
GO:0009812	Flavonoid metabolic process	489	98	48.4	9.80E-12
GO:0046942	Carboxylic acid transport	317	72	31.4	1.50E-11
GO:0016310	Phosphorylation	5,363	669	531.0	1.70E-11
GO:0044699	Single-organism process	17,160	1,886	1699.1	2.30E-11
***PHYC*-regulated**					
GO:0006952	Defense response	1,209	43	19.0	7.70E-07
GO:0042592	Homeostatic process	1,321	43	20.8	7.20E-06
GO:0006468	Protein phosphorylation	3,908	94	61.6	2.10E-05
GO:0016310	Phosphorylation	5,363	121	84.5	2.20E-05
GO:0016045	Detection of bacterium	5	3	0.1	3.80E-05
GO:0098543	Detection of other organism	5	3	0.1	3.80E-05
GO:0098581	Detection of external biotic stimulus	8	3	0.1	0.00021
GO:0065008	Regulation of biological quality	2,033	53	32.0	0.00025
GO:0048878	Chemical homeostasis	621	22	9.8	0.00041
GO:*0006879*	Cellular iron ion homeostasis	42	5	0.7	0.0005
***PHYB*/*PHYC*-regulated**					
GO:0006952	Defense response	1,209	30	11.1	9.10E-07
GO:0006468	Protein phosphorylation	3,908	62	35.7	1.10E-05
GO:0006879	Cellular iron ion homeostasis	42	5	0.4	4.00E-05
GO:0016310	Phosphorylation	5,363	76	49.0	5.10E-05
GO:0055072	Iron ion homeostasis	92	6	0.8	0.00021
GO:0050896	Response to stimulus	7,059	90	64.5	0.0004
GO:0042592	Homeostatic process	1,321	25	12.1	0.00054
GO:0006950	Response to stress	3,927	54	35.9	0.00153
GO:0048872	Homeostasis of number of cells	7	2	0.1	0.0017
GO:0009812	Flavonoid metabolic process	489	12	4.5	0.00199

Table S3: Summary of RNA-seq reads and mapping rates from LD samples.

Genotype	Exp. Rep.	Bio. Rep.	Raw reads	Reads discarded		Trimmed reads	Uniquely mapped reads	
				N	%		N	%
**PHYB-WT**	**1**	1	38,193,836	2,291,251	6.0	35,902,585	18,977,319	52.9
		2	37,228,077	2,236,373	6.0	34,991,704	18,615,718	53.2
		3	50,793,083	2,966,158	5.8	47,826,925	25,410,013	53.1
		4	44,301,543	2,715,483	6.1	41,586,060	21,258,178	51.1
	**2**	1	53,475,278	3,983,069	7.5	49,492,209	26,092,448	52.7
		2	54,275,607	3,972,114	7.3	50,303,493	26,657,660	53.0
		3	57,095,077	4,313,269	7.6	52,781,808	27,415,963	51.9
		4	59,581,947	4,279,495	7.1	55,302,452	29,303,043	53.0
phyB-null	**1**	1	43,374,525	2,540,401	5.9	40,834,124	21,739,116	53.2
		2	47,201,620	2,731,653	5.8	44,469,967	23,537,045	52.9
		3	81,109,422	4,903,617	6.0	76,205,805	40,552,227	53.2
		4	47,250,183	2,769,089	5.9	44,481,094	23,788,301	53.5
	**2**	1	47,550,092	3,517,065	7.4	44,033,027	23,533,579	53.5
		2	50,880,434	3,783,852	7.4	47,096,582	25,126,305	53.4
		3	56,371,576	4,144,097	7.4	52,227,479	27,938,705	53.5
		4	51,113,770	3,679,790	7.2	47,433,980	25,437,698	53.6
**PHYC-WT**	**1**	1	40,263,724	4,609,655	11.5	35,654,069	18,918,318	53.1
		2	40,409,065	4,363,202	10.8	36,045,863	20,341,146	56.4
		3	42,605,342	4,775,194	11.2	37,830,148	20,222,651	53.5
		4	39,796,668	4,597,431	11.6	35,199,237	18,893,499	53.7
	**2**	1	37,994,979	3,339,800	8.8	34,655,179	18,184,337	52.5
		2	43,262,009	3,678,319	8.5	39,583,690	21,115,165	53.3
		3	41,619,486	3,613,203	8.7	38,006,283	20,202,760	53.2
		4	31,887,538	2,770,104	8.7	29,117,434	15,386,865	52.8
phyC-null	**1**	1	44,904,496	4,989,736	11.1	39,914,760	21,705,064	54.4
		2	46,776,974	5,163,917	11.0	41,613,057	22,393,932	53.8
		3	43,960,996	4,795,476	10.9	39,165,520	21,053,322	53.8
		4	42,873,364	4,595,730	10.7	38,277,634	20,718,521	54.1
	**2**	1	35,291,647	2,998,278	8.5	32,293,369	17,279,830	53.5
		2	36,712,107	3,016,466	8.2	33,695,641	17,747,367	52.7
		3	45,656,274	3,815,739	8.4	41,840,535	22,551,769	53.9
		4	38,922,327	3,391,092	8.7	35,531,235	13,034,303	36.7
Mean			46,022,908	3,729,379	8.2	42,293,530	22,347,880	52.8

Table S4 – Top 10 significant enriched GO terms among genes differentially regulated by both *PHYB* and *PHYC* under different photoperiod conditions.

GO ID	Term	Annotated	Significant	Expected	*P*
***PHYB-PHYC* regulated – SD specific**					
GO:0006468	Protein phosphorylation	5,201	88	41.65	5.10E-12
GO:0016310	Phosphorylation	7,036	105	56.35	4.60E-11
GO:0019725	Cellular homeostasis	845	23	6.77	4.50E-07
GO:0006952	Defense response	1,563	33	12.52	5.10E-07
GO:0042592	Homeostatic process	1,597	33	12.79	8.10E-07
GO:0006796	Phosphate-containing compound metabolic process	9,005	109	72.12	2.00E-06
GO:0006793	Phosphorus metabolic process	9,117	109	73.02	3.60E-06
GO:0045454	Cell redox homeostasis	356	13	2.85	7.20E-06
GO:0006464	Cellular protein modification process	8,493	99	68.02	3.60E-05
GO:0036211	Protein modification process	8,493	99	68.02	3.60E-05
***PHYB-PHYC* regulated – LD specific**					
GO:0048586	Regulation of LD photoperiodism, flowering	30	2	0.02	0.00015
GO:0042752	Regulation of circadian rhythm	31	2	0.02	0.00016
GO:2000028	Regulation of photoperiodism, flowering	41	2	0.02	0.00029
GO:2000243	Positive regulation of reproductive process	42	2	0.03	0.0003
GO:0048574	LD photoperiodism, flowering	43	2	0.03	0.00032
GO:0005982	Starch metabolic process	224	3	0.14	0.00034
GO:0048571	LD photoperiodism	45	2	0.03	0.00035
GO:0010218	Response to far red light	52	2	0.03	0.00046
GO:0007623	Circadian rhythm	53	2	0.03	0.00048
GO:0048511	Rhythmic process	57	2	0.03	0.00056
***PHYB-PHYC* regulated – SD and LD**					
GO:0050789	Regulation of biological process	10,086	17	5.9	3.20E-06
GO:0009889	Regulation of biosynthetic process	5,573	12	3.26	2.40E-05
GO:0065007	Biological regulation	11,745	17	6.88	2.90E-05
GO:0006355	Regulation of transcription, DNA-templated	5,066	11	2.97	5.80E-05
GO:0080090	Regulation of primary metabolic process	6,135	12	3.59	6.40E-05
GO:1903506	Regulation of nucleic acid-templated transcription	5,113	11	2.99	6.40E-05
GO:2001141	Regulation of RNA biosynthetic process	5,113	11	2.99	6.40E-05
GO:0051252	Regulation of RNA metabolic process	5,162	11	3.02	7.00E-05
GO:0019219	Regulation of nucleobase-containing compound metabolic process	5,317	11	3.11	9.10E-05
GO:0048573	Photoperiodism, flowering	157	3	0.09	0.00011

Table S5: Selected genes regulated by both *PHYB* and *PHYC* in both SD and LD photoperiods. Fold change between WT and respective *phy*-null mutants are presented. The values for LD are the mean fold-change in expression between both experimental replicates. a - *FT-A1* and *FT-B1* expression was zero in the *phy*-null mutants in these comparisons.

		Fold change in expression			
		Short days		Long days	
**Gene ID**	**Annotation**	**WT/*phyB*-null**	**WT/*phyC*-null**	**WT/*phyB*-null**	**WT/*phyC*-null**
TraesCS7A01G115400	*FT-A1*	- [a]	168.88	- [a]	- [a]
TraesCS7B01G013100	*FT-B1*	- [a]	286.72	927.23	481.46
TraesCS3A01G143100	*FT-A2*	38.02	78.31	119.44	16.64
TraesCS1B01G351100	*FT-B3*	14.44	14.18	3.56	7.52
TraesCS5A01G391700	*VRN-A1*	3.09	2.66	15.64	7.96
TraesCS5B01G396600	*VRN-B1*	70.61	52.50	113.15	73.89
TraesCS2A01G261200	*FUL-A2*	92.75	644.50	800.56	127.00
TraesCS2A01G174300	*FUL-A3*	351.75	369.78	740.74	-
TraesCSU01G196100	*PPD-B1*	25.37	9.95	322.38	35.17
TraesCS6A01G273200	MYB	21.12	8.30	43.09	2.89
TraesCS6B01G300600	MYB	7.99	5.11	4.05	3.63
TraesCS1A01G220300	CONSTANS-like	3.36	2.90	3.82	2.48
TraesCS3B01G365300	VQ-motif protein	0.19	0.31	0.01	0.08
TraesCS5A01G520200	*TaFLC-A2*	0.35	0.23	0.12	0.18
TraesCS4B01G351500	*TaFLC-B2*	0.18	0.11	0.01	0.02
TraesCS3A01G435000	*TaFLC-A4-1*	0.22	0.06	0.14	0.12
TraesCS2A01G427200	*WCOR15*	0.03	0.11	0.01	0.05

Table S6: Selected genes regulated by both *PHYB* and *PHYC* in SD photoperiods but not LD photoperiods. Fold change between WT and respective *phy*-null mutants are presented as the mean fold-change between both experimental replicates. a - *FT-A4* expression was zero in the *phy*-null mutants in these comparisons.

		**Fold change in short days**	
**Gene ID**	**Annotation**	**WT/*phyB*-null**	**WT/*phyC*-null**
TraesCS3B01G162000	*FT-B2*	24.89	12.53
TraesCS2A01G132300	*FT-A4*	- [a]	6.80
TraesCS7B01G158900	*TaFLC-B1*	12.18	3.66
TraesCS2B01G378700	NF-YB	2.70	3.07
TraesCS7A01G233300	MYB_related	4.26	9.05
TraesCS7B01G131600	MYB_related	14.02	16.90
TraesCS5B01G054800	bHLH	5.55	2.59
TraesCS5B01G183700	WRKY	7.80	5.16
TraesCS3B01G129900	WRKY	13.83	6.20
TraesCS3B01G240200	WRKY	17.62	6.85
TraesCS7B01G249700	WRKY	123.51	205.05
TraesCS3B01G199000	WRKY	2.38	2.94
TraesCS5B01G183800	WRKY	4.22	2.92
TraesCS6A01G146900	WRKY	4.96	2.58
TraesCS1B01G374900	WRKY	5.07	2.47
TraesCS3A01G347500	WRKY	5.51	2.87
TraesCS1A01G334400	GA-2oxidase	0.05	0.13
TraesCS6A01G143900	BBox	0.06	0.17
TraesCS2A01G100600	G2-like	0.22	0.40
TraesCS3A01G274900	GATA	0.55	0.46
TraesCS1B01G335100	CBF	0.16	0.17
TraesCS5A01G311100	CBF	0.19	0.37
TraesCS5A01G310800	CBF	0.47	0.18
TraesCS5A01G310700	CBF	0.52	0.33

Table S7: Selected genes regulated by both *PHYB* and *PHYC* in LD photoperiods but not SD photoperiods. Fold change between WT and respective *phy*-null mutants are presented as the mean fold-change between both experimental replicates.

		Fold change in long days	
**Gene ID**	**Annotation**	**WT/*phyB*-null**	**WT/*phyC*-null**
TraesCS3A01G116300	*GIGANTEA-A*	2.35	3.21
TraesCS3B01G135400	*GIGANTEA-B*	2.59	3.99
TraesCS6B01G315400	CO-like	0.42	0.23
TraesCS7B01G115200	SPL	0.24	0.25
TraesCS3A01G107200	*TZF-A1*	0.09	0.18

Table S8: *PHYB*- and *PHYC*-regulated splicing-related genes. Mean TPM and fold-change for each genotype was calculated from four biological replicates in SD samples, and eight biological replicates for LD samples.

***PHYB* regulated under SD**		**TPM**		
**Gene ID**	**Annotation**	**Wild-type SD**	***phyB*-null_SD**	**Fold-change**
TraesCS3B01G157400	Pre-mRNA-splicing factor of RES complex protein	6.4	3.4	1.9
TraesCS1B01G125800	Pre-mRNA-splicing factor cwc26	10.0	2.3	4.3
TraesCS7B01G194600	Pre-mRNA-splicing factor SLU7	6.6	3.3	2.0
TraesCS5B01G017300	Splicing factor 3B subunit 1	0.2	0.9	0.2
TraesCS7B01G450300LC	Splicing factor 3B subunit 4	0.0	2.0	-
TraesCS2B01G720700LC	Splicing factor U2AF small subunit A	0.1	3.2	0.0
TraesCS4B01G209600	SR-rich pre-mRNA splicing activator	4.4	1.7	2.6
TraesCS4A01G094900	SR-rich pre-mRNA splicing activator	25.2	4.9	5.1
TraesCS2A01G122400	Splicing factor U2AF, large subunit	3.0	1.3	2.3
TraesCS7A01G075100	Splicing factor U2AF large subunit A	0.2	0.0	17.9
***PHYC*-regulated under SD**		**TPM**		
**Gene ID**	**Annotation**	**Wild-type SD**	***phyC*-null SD**	**Fold-change**
TraesCS1B01G125800	Pre-mRNA-splicing factor cwc26	10.0	2.2	4.6
TraesCS2A01G646800LC	Pre-mRNA-processing-splicing factor 8	0.1	1.1	0.1
TraesCS1B01G416400	Pre-mRNA-processing-splicing factor 8	3.1	0.2	15.6
TraesCS1B01G130200	Arginine/serine-rich splicing factor	0.2	1.8	0.1
TraesCS5B01G017300	Splicing factor 3B subunit 1	0.2	0.6	0.3
TraesCS7B01G450300LC	Splicing factor 3B subunit 4	0.0	2.7	-
TraesCS7B01G384700LC	Splicing factor family-like	0.5	0.0	-
TraesCS5A01G366000	Splicing factor U2AF large subunit	0.0	0.2	0.2
TraesCS2B01G720700LC	Splicing factor U2AF small subunit A	0.1	2.1	0.0
***PHYB*-regulated under LD**		**TPM**		
**Gene ID**	**Annotation**	**Wild-type LD**	***phyB*-null LD**	**Fold-change**
TraesCS2A01G122400	Splicing factor U2AF, large subunit	3.5	1.5	2.3
TraesCS3A01G086900	Splicing factor	5.8	9.8	0.6
TraesCS4A01G399500	Pre-mRNA-splicing factor CWC22-like protein	3.0	9.1	0.3
TraesCS3A01G426500	Lysine ketoglutarate reductase trans-splicing-like protein	14.2	7.0	2.0
TraesCS5A01G189600	Pre-mRNA-processing-splicing factor 8	0.9	1.6	0.6
TraesCS3B01G293100	Arginine/serine-rich splicing factor	9.3	11.6	0.8
TraesCS3A01G260000	Arginine/serine-rich splicing factor	11.7	18.4	0.6

TraesCS4A01G091300	RNA-binding protein	37.6	53.9	0.7
TraesCS1B01G244200	Splicing factor-like protein	4.3	6.5	0.7
TraesCS5A01G267500	Splicing factor U2AF small subunit A	19.9	34.7	0.6

***PHYC*-regulated under LD**		**TPM**		
**Gene ID**	**Annotation**	**Wild-type LD**	***phyC*-null LD**	**Fold-change**
TraesCS1B01G130200	Arginine/serine-rich splicing factor	0.1	0.5	0.2
TraesCS1B01G615200LC	Splicing factor 3B subunit 5	2.1	11.7	0.2
